# Isolation and Characterization of Bacteriophages That Infect *Citrobacter rodentium*, a Model Pathogen for Intestinal Diseases

**DOI:** 10.3390/v12070737

**Published:** 2020-07-08

**Authors:** Carolina M. Mizuno, Tiffany Luong, Robert Cederstrom, Mart Krupovic, Laurent Debarbieux, Dwayne R. Roach

**Affiliations:** 1 Department of Microbiology, Institut Pasteur, 75015 Paris, France; carolina.mizuno@gmail.com (C.M.M.); mart.krupovic@pasteur.fr (M.K.); laurent.debarbieux@pasteur.fr (L.D.); 2 Department of Biology, San Diego State University, San Diego, CA 92182, USA; tluong4407@sdsu.edu (T.L.); robbycederstrom@yahoo.com (R.C.); 3 Viral Information Institute, San Diego State University, San Diego, CA 92182, USA

**Keywords:** phages, phage therapy, hypoxic, resistance, *Vectrevirus*, vB_CroP_CrRp3, vB_CroP_CrRp10

## Abstract

Enteropathogenic *Escherichia coli* (EPEC) is a major pathogen for diarrheal diseases among children. Antibiotics, when used appropriately, are effective; however, their overuse and misuse have led to the rise of antibiotic resistance worldwide. Thus, there are renewed efforts into the development of phage therapy as an alternative antibacterial therapy. Because EPEC in vivo models have shortcomings, a surrogate is used to study the mouse pathogen *Citrobacter rodentium* in animal models. In this study, two new phages CrRp3 and CrRp10, which infect *C. rodentium,* were isolated and characterized. CrRp3 was found to be a new species within the genus *Vectrevirus,* and CrRp10 is a new strain within the species *Escherichia virus Ime09*, in the genus *Tequatrovirus.* Both phages appear to have independently evolved from *E. coli* phages, rather than other Citrobacter spp. phages. Neither phage strain carries known genes associated with bacterial virulence, antibiotic resistance, or lysogeny. CrRp3 is more potent, having a 24-fold faster adsorption rate and shorter lytic cycle when compared to the same properties of CrRp10. However, a lysis curve analysis revealed that CrRp10 prevented growth of *C. rodentium* for 18 h, whereas resistance developed against CrRp3 within 9 h. We also show that hypoxic (5% oxygen) conditions decreased CrRp3 ability to control bacterial densities in culture. In contrast, low oxygen conditions did not affect CrRp10 ability to replicate on *C. rodentium*. Together, CrRp10 is likely to be the better candidate for future phage therapy investigations.

## 1. Introduction

Diarrheal diseases continue to be one of the foremost public health issues globally, responsible for more than 1.6 million deaths each year [1]. While mortality rates have reduced substantially (by 34% among children and by 21% among all people) over the last two decades, the incidence of diarrhea has not reduced nearly as much (10% reduction in children and 6% reduction overall) [1]. Enteropathogenic *Escherichia coli* (EPEC) is a major cause of diarrhea morbidity and mortality among children < 5 years [1,2]. When used appropriately, antibiotics can reduce the severity and duration of diarrheal diseases; however, the overuse and misuse of antibiotics in the treatment of diarrhea has led to an alarming increase in antibiotic resistance (AMR) globally [1,2,3]. These observations illustrate that diarrhea mortality is largely avoidable and renewed efforts to reduce disease the burden with non-antibiotic strategies are urgently needed.

Mouse models for the study of EPEC diseases have had limited utility, because gastrointestinal microbiota defends well against human *E. coli* infections [4]. Consequently, most mouse models of *E. coli* gastrointestinal diseases require substantial antibiotic pre-treatment to reduce resident bacteria [5,6,7,8]. The preferred surrogate for EPEC diseases is the mouse-restricted pathogen *Citrobacter rodentium* [9]. Both EPEC and *C. rodentium* cause attaching and effacing (A/E) lesions, and share the same pool of locus of enterocyte effacement (LEE)-encoded and non-LEE-encoded effector proteins to subvert and modulate gut epithelial barrier properties [9,10]. Importantly, without the aid of antibiotics, *C. rodentium* causes pathologies that are indistinguishable from those observed in the human EPEC gut infection [11].

Bacteriophages (phages) are one of the foremost antibacterial agents under development and in clinical testing for treating life-threatening AMR pathogens [12,13], with several recent successful compassionate use phage therapy cases (for examples, see [14,15,16]). Phages are highly abundant and ubiquitous viruses that only infect and kill bacteria. Other promising features of phages include a narrow host range, self-replication at sites of infection, disruption of biofilms, and potential synergy with antibiotics [13,17,18]. Traditionally, virulent double-stranded DNA tailed phages that carry out a strictly lytic infection cycle are considered the ideal viral type for human therapeutic applications [12,13]. However, only four virulent phages that infect *C. rodentium* have been described to date, including phiCR1 [19], R18C [20], CR8 and CR44b [21]. In addition, *C. rodentium* phage therapy animal models have not been explored.

In this study, we characterize two new virulent phages, CrRp3 and CrRp10, which infect *C. rodentium*. Using genome sequence annotations, phenotypic susceptibility under normoxic and hypoxic environments, host cross-genera infection ranges, and phylogenetic analysis, we assess the subtle feature differences that may improve the efficacy of future therapeutic evaluation in an A/E diarrheal disease mouse model.

## 2. Materials and Methods

### 2.1. Strains and Culturing

*C. rodentium* strain ICC180 [22] was grown in Luria–Bertani (LB) broth under both normoxic (atmospheric 21% O_2_ and 0.04% CO_2_) and hypoxic (5% O_2_ and 5% CO_2_) environments at 37 °C with orbital shaking. For hypoxia culturing, LB was preconditioned at 5% O_2_ and 5% CO_2_ for 2 days by using an incubator with O_2_ control filled with 5% CO_2_ and balanced with N_2_ (NBS Galaxy 48R) to remove dissolved oxygen in growth media. Agar (1.5%) was added to LB for solid growth medium.

### 2.2. Phage Isolation, Cultivation and Purification

*C. rodentium* phages were isolated from 0.25 μm syringe filtered (Sartorius, Göttingen, Germany) sewage sample collected from water treatment plants around Paris, France. Individual plaque-forming units were isolated from a 10 μL sample spread over LB agar Petri plate, overlaid with 2 mL of mid-log growing ICC180 under normoxic conditions. After overnight incubation, a sterile Pasteur pipette was used to select PFU with different morphologies and phages enriched by culturing with ICC180 in fresh LB. PFU selection and enrichment was repeated 5 times. Final phage lysates were sterilized by high-speed centrifugation, 0.2 µm filtration, further purified by cross-flow filtration (CFF), and cesium chloride (CsCl) density gradient ultracentrifugation, using a methodology previously described [23]. Lastly, phages were stored in phosphate buffer saline (PBS).

### 2.3. Transmission Electron Microscopy

To visualize viral morphology, purified phages in TN buffer (10 mM Tris-Cl, 10 mM NaCl) were loaded onto carbon-coated copper grids and negatively stained with 2% (wt/vol) uranyl acetate for 30 s. Then, phages were visualized using a FEI Tecnai 120 Biotwin transmission electron microscope (TEM) (FEI, Hillsboro, OR, USA), at an accelerating voltage of 120 kV, and an Orius 1000 digital camera (Gatan, Pleasanton, CA, USA) recorded the micrographs.

### 2.4. DNA Extraction and Genome Sequencing

Genomic DNA was extracted from sterile DNase and RNase pretreated purified phages by a phenol-chloroform extraction, as previously described [24]. Sequencing libraries with single index were prepared using the NEBNext DNA library prep kit (New England BioLabs, Ipswich, MA, USA) and then sequenced on the Illumina MiSeq sequencing platform (Illumina, San Diego, CA, USA) with paired-end 300 nucleotide reads. Raw reads were trimmed by FastQC v10.1 (www.bioinformatics.babraham.ac.uk/projects/fastqc/) and de novo assembled using the CLC Genomics Assembler (Galaxy Version 4.4.2).

### 2.5. Genome Annotations, Alignments, and Phylogenetic Analyses

Annotation of the specific function of ORFs was conducted using rapid annotations of subsystems technology (RAST) [25]. The presence of transfer RNA (tRNA)-encoding genes was determined using the tRNAscan-SE database [26], and protein-coding genes were predicted using Prodigal [27]. The additional annotation of genes was done manually on the HHPRED server [28], and compared against the NCBI NR, COG [29], and TIGRfam [30] databases. A comparative analysis among related complete genomes was performed using tBLASTx or BLASTN [31]. A computational analysis of toxins, antibiotic resistance, and bacterial virulence factors was done with Abricate (https://github.com/tseemann/abricate), which uses ensemble methods to compare sequences to the most up-to-date databases, including the Comprehensive Antibiotic Resistance Database [32], ResFinder [33], NCBI’s AMRFinder [34], Antibiotic Resistance Gene-ANNOTation [35], and the virulence factor database [36]. Phylogenetic relationships of phages CrRp3 and CrRp10 were determined using VICTOR [37], and pairwise comparisons of the nucleotide sequences were conducted using the Genome-BLAST Distance Phylogeny (GBDP) method [38], under settings recommended for prokaryotic viruses [37]. GBDP phylogenetic relationships were constructed from related *Citrobacter, Escherichia* and *Synechococcus* podoviruses and myoviruses identified by Blast (downloaded from GenBank 12/21/2017; Table S4). The resulting intergenomic distances (100 replicates each) were used to infer a balanced minimum evolution tree with branch support via FASTME, including SPR post processing [39]. The trees were rooted with *Synechococcus* phages (outgroup) and visualized with FigTree V1.4.3 (http://tree.bio.ed.ac.uk).

### 2.6. Phage Adsorption and Growth

A similar protocol [40] was adopted to determine phage adsorption rate. Briefly, 1 mL of phages was mixed with 2.5 × 10^8^ colony forming units (CFU) mL^−1^ of bacteria in 9 mL at a multiplicity of infection (MOI) of 0.001. The mixture was incubated at 37 °C with shaking and samples withdrawn each 1 min for titration. In addition, we performed a one-step growth curve procedure as described previously [41], with some modifications. Briefly, 1 mL of phages was added to 2.5 × 10^8^ CFU mL^−1^ of *C. rodentium* in 9 mL LB, with no divalent cation supplementation, at a MOI of 0.01. At 2 min intervals, two 100 µL samples were taken, with one used to enumerate free phages, and the second was treated with 10% chloroform to release intracellular phages. At 6 min, a 1000x dilution in 100 mL of fresh broth was performed to avoid any potential future re-infection by newly produced phages. Samples were serial diluted in microtiter plates and PFU counted on LB agar seeded with lawns of bacteria at OD_600_ 0.2. Titer comparison between chloroform-treated and non-treated samples was used to estimate eclipse and latent periods, respectively. Adsorption and one-step growth analyses were repeated four times.

### 2.7. Phage Lysis Curves

*C. rodentium* strain ICC180 from an overnight culture was used to inoculate fresh LB to create an exponentially growing population with an optical density (OD_600_) of ~0.5. To maintain hypoxia, bacteria was grown in LB that was preconditioned overnight in 5% O_2_ and 5% CO_2_ chamber. Cells were then washed twice by centrifugation at 6000× *g* for 10 min and then resuspended in chilled H_2_O. Microplate wells were filled with 100 µL of 2 times LB concentrate (preconditioned if required) before adding washed cells to give a total of 2 × 10^6^ CFU. Phages were added at different MOIs and sterile water added for a total volume of 200 µL. Microplates were immediately placed in a Clariostar microplate reader (BMG Labtech, Cary, NC, USA) and OD_600_ measured every 6 min for 18 h. Microplate reader O_2_ and CO_2_ levels were regulated with an atmospheric control unit (BMG labtech).

### 2.8. Phage Host Range

We evaluated phage host ranges using a spot test of 4 µL of 10^7^ PFU onto 32 bacterial strains (listed in Table 3), seeded on agar at OD_600_ 0.22, followed by overnight incubation at 37 °C. Bacteria tested included 27 *Escherichia coli* strains, *Erwinia carotovora* CFBL2141 [42], *Rouxiella chamberiensis* [43], *Serratia marcescens* DB11 [44] and SM365 [45]. For comparison, the host ranges were also determined for *E. coli* phages LF82_P10 [46], LM33_P1 [47], AL505_P2 [48], and CLB_P2 [49], 536_P7 [50].

### 2.9. Statistics

Data are shown as means ± standard deviation (SD). Statistical analyses were performed using Prism v5 (GraphPad, San Diego, CA, USA). A Student’s *t*-test or one-way analysis of variance with Tukey’s multiple-comparison test was used to determine differences between two or multiple groups, respectively. A *p* < 0.05 was considered significant.

### 2.10. Accession Numbers

The complete genome sequences of vB_CroP_CrRp3 (CrRp3) and vB_CroM_CrRp10 (CrRp10) are deposited in NCBI GenBank with accession numbers MG775042 and MG775043, respectively.

## 3. Results

### 3.1. Isolation, Morphology, Sequencing, and Genome Annotation of CrRp3

**Figure 1:**
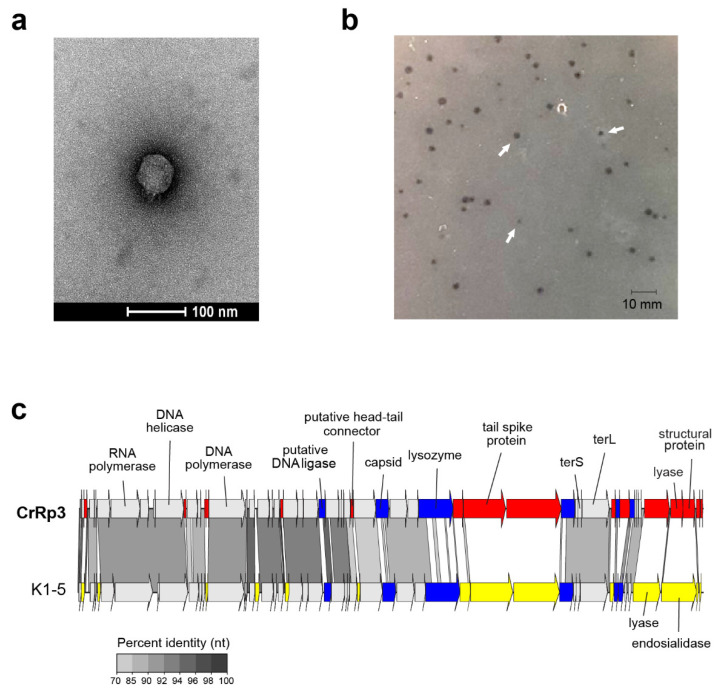
Morphology, genome organization and gene functional comparison of CrRp3. (**a**) Electron micrographs of CrRp3 negatively stained with uranyl acetate. (**b**) Plaque forming units (highlighted by arrows) produced by CrRp3 on lawns of *C. rodentium* ICC180 after 24 h of growth. (**c**) Genome alignment of CrRp3 and its closest taxonomic relative *E. coli* phage K1-5. Genes are colored according to nucleotide sequence homology, with genes exclusive to CrRp3 labeled red, genes exclusive to K1-5 labeled yellow, and homologous genes with <70% identity labeled blue.

The novel phage CrRp3 (formal name: vB_CroP_CrRp3), which infects *C. rodentium* strain ICC180, was isolated from sewage. Figure 1a shows that CrRp3 virion has a typical podovirus morphology, with an isometric head, likely icosahedral, and a short tail. Plaques produced by CrRp3 on ICC180 were of 2.36 ± 0.46 mm^2^, mean ± 95% confidence interval (Figure 1b).

Mapping next generation sequence reads showed that CrRp3 has a terminally repetitive dsDNA genome of 44.3 kb, with 54 predicted coding sequences (CDSs) (Figure 1c and Table 1). However, only 19 (35%) could be assigned a putative function (Table S1). Interestingly, CrRp3 has a GC content of 45%, which is 10% lower than that in its host *C. rodentium,* which has a GC content of 54.5%. The genome does not encode proteins with identifiable sequence homology to known lysogeny-associated proteins, suggesting that CrRp3 is virulent with a strictly lytic lifecycle. In addition, CrRp3 genome does not carry recognizable virulence-associated genes or antibiotic resistance genes.

At the time of analysis, the closest taxonomic relative of CrRp3 was the *E. coli* phage K1-5 from the *Vectrevirus* genus in the recently created family *Autographivirinae*. The two phages share both gene content and genome organization with 90% (over 76% of the genome) and 77% (over 75% of the genome) pairwise nucleotide identity, respectively (Figure 1c). Nearly half of the CrRp3 gene products have the best hits with K1-5 genes, including the DNA and RNA polymerases, DNA ligase and the major capsid protein, as well as many hypothetical proteins (Table S3). Accordingly, CrRp3 is a tentative new species within the genus *Vectrevirus* in the family *Autographiviridae* [58]. For most phage genera, >95% DNA sequence identity is used by the International Committee on Taxonomy of Viruses (ICTV), as the species demarcation criterion [58]. Gene products unique to CrRp3 include the head-tail connector protein, endolysin, tailspike protein, lyase, minor structural protein, and several other proteins with unknown functions (Figure 1c and Table S1).

### 3.2. Morphology and Genome of Phage CrRp10

**Figure 2:**
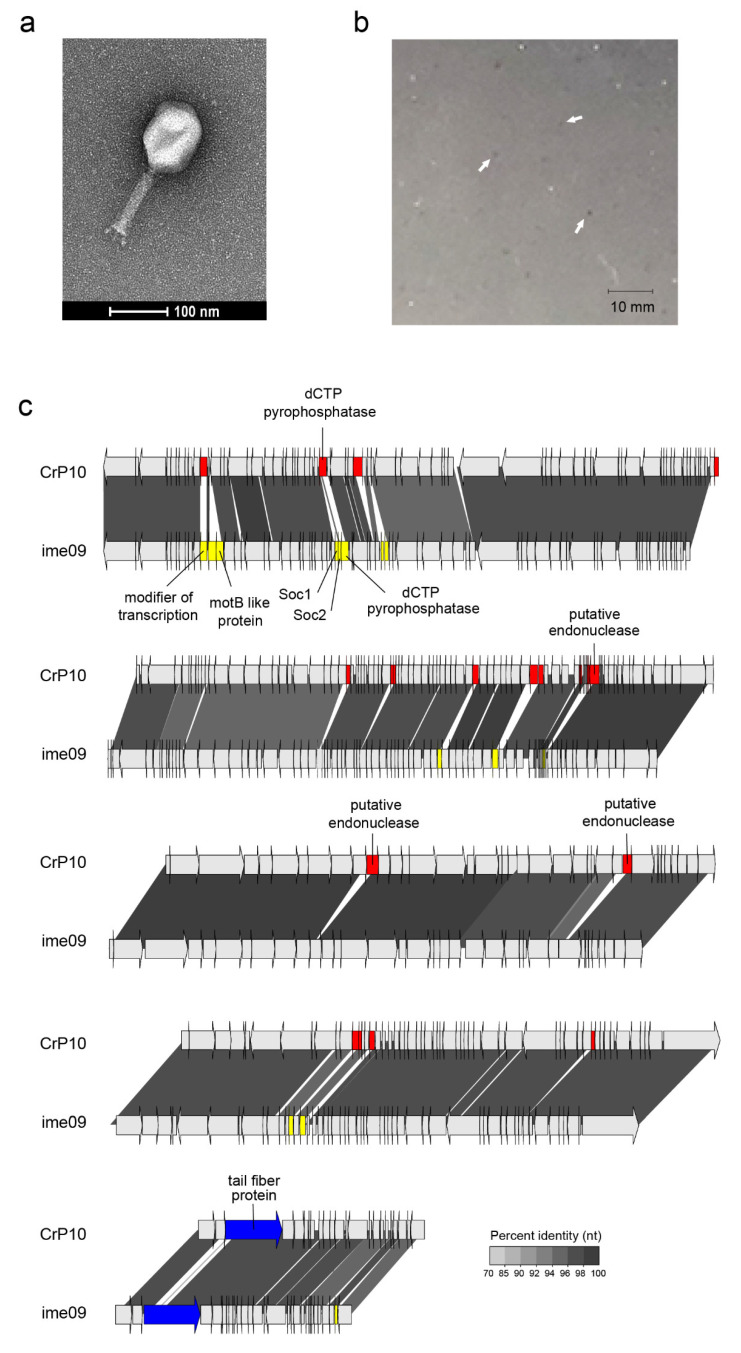
Morphology, genome organization and gene functional comparison of CrRp10. (**a**) Electron micrographs of CrRp10 negatively stained with uranyl acetate. (**b**) Plaque forming units (highlighted by arrows) on lawns of *C. rodentium* ICC180 after 24 h or growth. (**c**) Genome alignment of CrRp10 and its closest taxonomic relative *E. coli* phage ime09. Genes are colored according to nucleotide sequence homology, with genes exclusive to CrRp10 labeled red, genes exclusive to ime09 labeled yellow, and homologous genes with <70% identity labeled blue.

The second isolated phage, *C. rodentium* phage CrRp10 (formal name: vB_CroM_CrRp10) displays an elongated (prolate) icosahedral head connected to a long tail covered with a discernable sheath (Figure 2a). These features are characteristic of T4-like myoviruses. Plaques produced by CrRp10 on ICC180 were of <1 mm^2^ (Figure 2b). This suggests that phage strain has a significant effect on the plaque size, when compared to plaques produced by CrRp3 (Figure 1b vs. Figure 2b).

Genome sequencing showed that CrRp10 has a large circularly permuted dsDNA genome of 171.5 kb with 267 CDSs, and harbors 10 tRNA genes (Figure 2c and Table 1). Only 133 (~50%) of CDS have putative functions (Table S2). Similar to CrRp3, CrRp10 has a GC content of 35.5%, which is 20% lower than that of *C. rodentium.* The genome does not carry sequence homology to lysogeny-associated genes, which suggests that CrRp10 is a virulent phage. The genomes also exhibit no sequence homologies to known virulence-associated or antibiotic resistance genes.

At the time of analysis, the closest taxonomic relative of CrRp10 was the *E. coli* phage Ime09, which belongs to the *Tequatrovirus* genus in the subfamily *Tevenvirinae* (Figure 2c and Table S3). They share significant synteny, with 98% nucleotide identity over the complete length, and thus, CrRp10 is considered a new strain. However, little is known about phage Ime09, with details restricted to genomic analysis [59]. CrRp10 has divergence from Ime09 within its tail fiber gene, with 3% amino acid dissimilarity over 80% of the corresponding protein. Another notable feature of the CrRp10 genome is what appears to be a recombination event, which resulted in the gain of dUTPase with high sequence similarity to that encoded by phage e11/2. Interestingly, phage e11/2 infects the enterohemorrhagic *E. coli* (EHEC); also an A/E pathogen [60]. Other recombination events appear to have added several putative endonucleases, with high similarity to homologs in other related Enterobacteriaceae phages (Figure 2c and Table S2).

### 3.3. Comparison of Citrobacter Phage Proteins

We performed amino acid (AA) sequence alignments to compare proteins among phages that infect *C. rodentium* and other *Citrobacter* species (Figure S1). Surprisingly, CrRp3 has <40% AA homology to either *C. rodentium* phages (CR8 and CR44b) or *C. freundii* phages phiCFP-1 and Sh4, with the exception of homology between a putative lyase (68%) and minor structural protein (75%) of *Citrobacter* phage CR8 (Figure S1a and Table S3). Several CrRp10 proteins exhibit low AA homology to the *C. rodentium* phage Moon, and even less homology with *C. freundii* phage CfP1 (Figure S1b and Table S3).

### 3.4. CrRp3 Is Faster at Infecting a Host Cell, but Has a Smaller Burst Size

With an excess of host cells, the estimated adsorption rates for phages CrRp3 and CrRp10 are 3.5 ± 3.2 × 10^−10^ and 8.52 ± 2.8 × 10^−11^ mL^−1^ min^−1^, respectively (Table 2). That is, when compared, CrRp3 would ‘infect a host’ ~24x faster than CrRp10. This also suggests that these two phages have different cell surface binding receptors. In a well-mixed culture, the CrRp3 replication cycle (latent period) takes approximately 15 min (Table 2 and Figure S2). CrRp10 however, had a slightly longer latent period of about 17 min. The eclipse periods could not be determined because *C. rodentium* appears to be resistant to chloroform lysis (Figure S2). The shorter replication cycle for CrRp3 correlated had a reduced burst size of 43 phage particles per cell, compared to CrRp10 that produced a burst of 85 phage particles (Table 2).

### 3.5. Hypoxia Reduces Lysis at Low MOIs but Not Resistance

**Figure 3:**
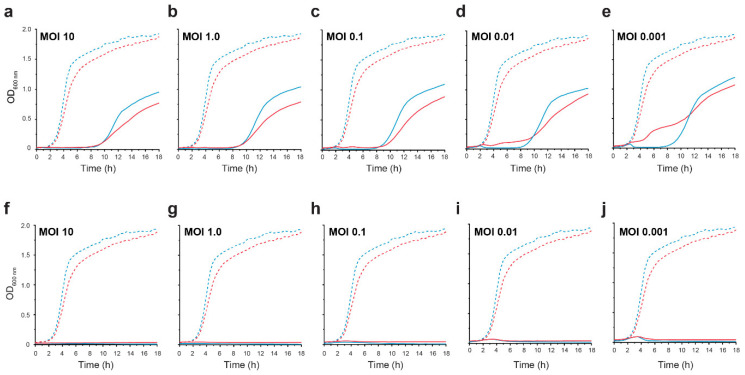
CrRp3 or CrRp10 lysis curves of *C. rodentium* under normoxic and hypoxic conditions. Growth curves of *C. rodentium* ICC180 infected with CrRp3 (**a**–**e**) or CrRp10 (**f**–**j**) at MOI ranging between 10–0.001 (solid line) or no phage control (dashed line). Bacteria were growth under either 21% (blue line) or 5% (red line) oxygen and 5% CO_2_ conditions. N = 6 per condition.

Healthy gastrointestinal luminal oxygen levels decreased from 7% (58 mmHg) in the stomach to 0.5% in the colon, and mucosa oxygen levels ranged between 2–6% oxygen [61]. Both CrRp3 and CrRp10 were able to reduce *C. rodentium* population densities compared to untreated controls at different MOIs under both normoxic (~21% O_2_) and hypoxic (5% O_2_) culturing conditions (Figure 3). Under normoxia, CrRp3 took less time than CrRp10 to reverse bacterial population growth (~2.5 h post treatment), and reduced bacterial density below OD limits of detection (LOD) within 3 h (Figure 3a). For CrRp10, the same results took 3.5 and 5.5 h, respectively (Figure 3b). Of these two phages, CrRp3 appears to be more ‘potent’. Under hypoxia, however, CrRp3 at MOIs <0.01 failed to reduce bacterial density below the LOD, whereas CrRp10 could (Figure 3). Indeed, hypoxia also dampened exponential bacterial growth (Figure 3) in the absence of phages, but final cell densities were similar after 18 h. In addition, the regrowth of *C. rodentium* occurred between 8–9 h after inoculation with CrRp3. In contrast, no observable bacterial regrowth occurred for CrRp10 after 18 h.

### 3.6. Host Range

Next, we tested the host range of the virulent phages CrRp3 and CrRp10, along with other representative phages against several bacterial strains (Table 3). In addition to their isolation strain of *C. rodentium*, CrRp3 could infect only the *E. coli* strain K-12, while CrRp10 displays a much broader host range, including K-12 and several pathotypes of *E. coli,* as well as the *E. carotovora* strain CFBP2141. Although the *E. coli* phage LF82_P10 [46] also exhibits a relatively broad host range, it cannot infect *C. rodentium*. Moreover, most of the *E. coli*, as well as the *Serratia marcescens* strains tested, were resistant to both CrRp3 and CrRp10.

### 3.7. Phage Phylogenetic Relationships

**Figure 4:**
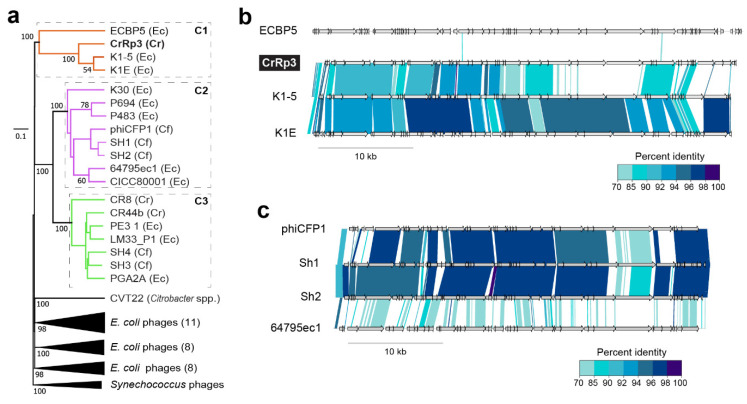
Phylogenetic relationships among *Citrobacter* and *Escherichia* podoviruses. (**a**) Tree assembled with complete sequences of podoviruses that infect *C. rodentium* (Cr), *C. freundii* (Cf) and related *E. coli* (Ec). The tree is rooted using *Synechococcus* phages and bootstrap values at nodes defining percent confidence of 100 replicates. Phylogenetic clades (C1–3) have strong bootstrap support of >70%. (**b**) Complete genome nucleotide alignment of members in clade 1, which includes CrRp3. (**c**) Genome alignments of members in C2, including related *Citrobacter* phages, Sh1, Sh2 and phiCFP1 that infect *C. freundii*.

**Figure 5:**
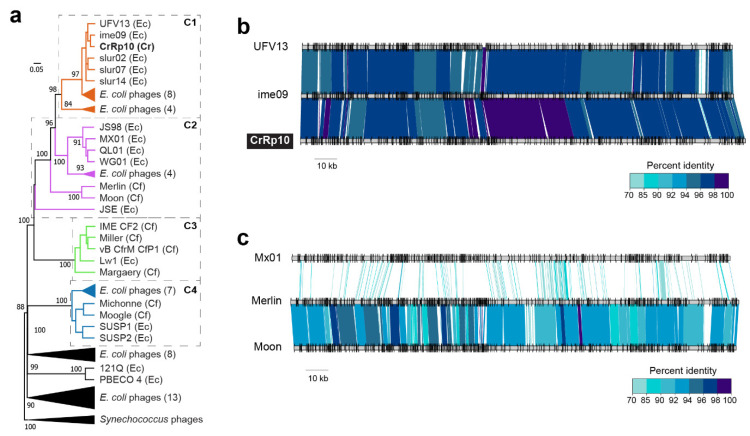
Phylogenetic relationships among *Citrobacter* and *Escherichia* myoviruses. (**a**) Tree assembled with complete genome sequences of myoviruses that infect *C. rodentium* myoviruses (Cr), *C. freundii* (Cf) and related *E. coli* (Ec). The tree is rooted using *Synechococcus* phages and bootstrap values at nodes defining percent confidence of 100 replicates. Phylogenetic clades (C1–4) have strong bootstrap support of >70%. (**b**) Complete genome nucleotide alignment of members in C1, which includes CrRp10. (**c**) Genome alignment of members in C2, including the closest related *Citrobacter* phages, Merlin and Moon, which infect *C. freundii*.

To investigate phylogenetic relationships among *Citrobacter* and *Escherichia* podoviruses including CrRp3, we constructed a genome-blast distance phylogeny tree using 37 complete genome nucleotide sequences from GenBank (Figure 5 and Table S4). We found that these phages display the heterogeneous clustering of closely related *Autographiviridae* into three distinct clades with maximal bootstrap support (Figure 4a). CrRp3 has closer relationships with podoviruses infecting *Escherichia* (Figure 4b) than those also infecting *C. rodentium* (CR8 and CR44b) (Figure 4a; see C1 vs. C3). Rather, CR8 and CR44b clusters with some phages that infect the human pathogen *C. freundii* (SH3 and SH4). Other *C. freundii* podoviruses (phiCFP1, SH1, and SH2) cluster in clade 2 (Figure 4c), and phage CVT2 branches separately. The latter was not unexpected, because CVT2 was isolated from the gut of termites with an uncharacterized *Citrobacter* species [51].

To determine the relationship of CrRp10 to other *Citrobacter* and *Escherichia Myoviridae*, we constructed a genome-blast distance tree using 69 complete genome sequences from GenBank (Figure 5 and Table S4), also showing heterogeneous clustering (Figure 5a and Table S3). CrRp10 clusters in a clade with several *E. coli* myoviruses (Figure 5). Other myoviruses that infect *C. freundii* cluster in clades 2–4. Within clade 2 (C2), *C. freundii* phages Merlin and Moon are further distantly related to 8 *Escherichia* phages (Figure 5c). In contrast, clade 3 (C3) is almost exclusively composed of *C. freundii* phages (IME CF2, Miller, CfP1, and Margaery).

## 4. Discussion

EPEC, EHEC, and the mouse pathogen *C. rodentium*, are all attaching/effacing pathogens and causative agents of diarrheal disease. In this study, we isolated and characterized two new phages, CrRp3 and CrRp10, which infect *C. rodentium*. We show that the podovirus CrRp3 is a tentative new species within the genus *Vectrevirus* in the family *Autographiviridae*, while the myovirus CrRp10 is a new strain within the *Tequatrovirus* genus in the family *Myoviridae.* Between CrRp3 and CrRp10, only 48% of their combined gene repertoire have assigned putative functions. Of the assignments, we found that neither phage harbor known genes associated with bacterial virulence or antibiotic resistance. The latter is consistent with previous findings that antibiotic resistance genes are rarely carried in phage genomes [62]. In addition, we show that CrRp3 and CrRp10 genomes lack identifiable integrase genes. This implies that both phages carry out strictly lytic replication cycles. Indeed, with more than 50% of genes having an unknown function, these findings emphasize the critical need for significant gene functional studies before any certainty that CrRp3 and CrRp10 do not carry harmful genes.

While these phages expand our knowledge of viral biodiversity, we found that neither CrRp3 nor CrRp10 genomes exhibit nucleotide sequence homology with other *C. rodentium* phages, including CR8 and CR44b from the genus *Caroctavirus* [21]. In addition, CrRp3 and CrRp10 are distantly related to phages that infect *C. freundii*, including members of the genera *Moonvirus* (Merlin, Miller, Moon) [52,53,54], *Mooglevirus* (Moogle, Michonne, Mordin) [55,56,63], *Tlsvirus* (Stevie) [57], and *Teetrevirus* (phiCFP-1, SH1, SH2, SH3, SH4, SH5) [64,65]. *C. freundii* can cause a variety of nosocomial acquired extraintestinal human diseases, such as the urinary tract, respiratory tract, and wound infections [66]. Thus, we show that CrRp3 and CrRp10 appear to have independently evolved from closely related *E. coli* phages, presumably because it was advantageous to expand the host range to infect *C. rodentium,* and as a result, occupy new niches. Petty et al. showed that the genome of *C. rodentium* exhibits several features typical of recently passing through an evolutionary bottleneck, including several large-scale genomic rearrangements and functional gene loss in the core genomic regions [67,68,69]. This led the authors to hypothesize that *C. rodentium* evolved from a human *E. coli* strain [69]. Our results strengthen this hypothesis by showing that phages that infect *C. rodentium* appear to have also evolved from phages that infect *E. coli*. Nonetheless, CrRp3 carries genes that have diverged significantly from presumably ancestral genes of *E. coli* K1-5-like phage, in particular, gene products responsible for receptor recognition (tail fibers) and cell lysis (endolysin). Interestingly, phage K1-5 exhibits two tail fiber genes, which allow it to be promiscuous between *E. coli* strains with different capsule compositions [70]. CrRp3 endolysin gene has homology to other *Citrobacter* phage endolysin genes. This suggests a mosaic genome structure driven by recombination events from diverse viruses. This is consistent with other autographiviruses that exhibit a high genetic identity, structure, and specific RNA polymerase, with the modest differences observed in genes implicated in adaptation to host constraints [71].

Lysis of bacterial cells provides insight into the dynamics between individual phages and their host bacteria. We found that CrRp3 appears to be the more ‘potent’ virus of the two. First, it was determined that CrRp3 would be 24x faster at infecting a host cell based on adsorption rates compared to CrRp10. Either this could be due to cell surface receptors for CrRp3 outnumbering receptors for CrRp10, or CrRp3 tail fibers have a higher affinity to a shared receptor. Second, in well-mixed cultures, CrRp3 took approximately 2 min less than CrRp10 to complete a single lytic replication cycle. This short cycle time correlated to CrRp3 reversing *C. rodentium* exponential growth sooner than CrRp10 (Figure 3). CrRp3 also exhibited, on average, a 51% reduced progeny burst compared to CrRp10′s burst size (Table 2). This raises the question of whether shorter replication cycles are favorable to higher phage progeny production to eliminate bacterial infections.

However, CrRp10 was more resilient to resistance. We found that CrRp10 did not allow the population growth of *C. rodentium* after 18 h (end of study), even at an initial MOI of 0.001 (i.e., 1 virion to 1000 cells) (Figure 3b). In contrast, *C. rodentium* exhibited regrowth after 9 h of co-incubation with CrRp3. Bacteria thwart phage attack through an arsenal of antiviral mechanisms targeting most steps of the phage replication cycle. The transition from phage-sensitive to phage resistant is often due to spontaneous chromosomal mutations that modify cell surface receptors for the phages [72]. Resistance mutations may be expected to impart a fitness cost because they target important biological functions in the cell [73]. This suggests that the mutation of the specific receptors used by CrRp10 to bind to bacterial hosts imposes much higher fitness costs than resistance to CrRp3′s receptors. Furthermore, second-site compensatory mutations did not lessen or alleviate the fitness costs associated with resistance to CrRp10. In relation to phage therapy, El Haddad et al. found that 7 out of 12 clinical studies confirmed that resistance had emerged during phage treatment [74]. Although the consequences of phage resistance in the clinic are under-studied, they could be the root cause of phage therapy clinical trial failures, for example [75]. Thus, selecting therapeutic agents like phage CrRp10, which was resilient to resistance in vitro, may lead to improvements in phage therapy efficacy.

To the best of our knowledge, this is the first investigation of phage infection under a controlled low-oxygen atmosphere (<10 mmHg); herein described as “physiologic hypoxia”. Hypoxia is a common feature during inflammation associated with bacterial infection and the intestinal luminal environment [61,76]. In addition, host intestinal epithelial cells maintain physiologic hypoxia by counter-current blood flow [77]. Enteropathogenic A/E pathogens secrete the virulence protein (effector) NleB, which alters the function of a master regulator of cellular O_2_ homeostasis, HIF-1α, thereby increasing O_2_ levels between 2–5% for glycolysis [77]. We show that 5% oxygen caused a marked delay in the ability of CrRp3 to create a bacterial population decline compared to infections under normoxia. With the higher concentration of oxygen, the physiologic conditions of the host bacterium may have been at a more favorable metabolic state for a higher productive phage burst or faster lytic cycle completions [78,79]. By contrast, enteric pathogens adapt to oxygen limitations by entering into a metabolically reduced state [80], which was observed as a slightly slower growth rate (Figure 3). The reduced growth rate may have affected phage-bacteria interactions and/or reduced the expression of phage receptors. This contrasts with our knowledge that other phages do not replicate in slow-growing bacteria, for example [81,82]. Our results warrant further investigations into the effects of hypoxia on phage-bacteria interactions.

Another criterion for the selection of therapeutic phages is the spectrum of bacterial species or strains lysed. We found that CrRp3 and CrRp10 exhibit polyvalence in hosts across genera within the *Enterobacteriaceae*. We show that, while CrRp3 produced plaques on lawns of *C. rodentium* and the non-pathogenic *E. coli* strain K12, CrRp10 also produced plaques on eight other *E. coli* strains (pathogenic and non-pathogenic) and the plant pathogen *E. carotovora*. In contrast, most phages are confined to a single host species and often to a subset of strains [47,83]. For example, the *C. rodentium* phage phiCR1 was unable to produce plaques on *E. coli* [19]. Considering the well-documented, collateral effects of broad-spectrum antibiotics, which are notorious for secondary outcomes such as antibiotic-associated diarrhea [84], a narrow phage spectrum may be advantageous during therapy. However, species specificity comes with inherent constraints. By selecting a phage that is limited to a single species or limited number of strains, treatment is likely to be less effective against multispecies or polystrain infections [85].

The overuse and misuse of antibiotics in the treatment of diarrhea have led to an alarming increase of AMR in diarrheagenic bacteria [1,2,3]. Phage therapy has been effective at reducing *E. coli* burden in the murine gut with antibiotic pretreatment [5,6,7,8]. Although *C. rodentium* is widely used as an exemplary in vivo model system for gastrointestinal bacterial diseases [9,86], there are no reports using this species for phage therapy development. Prior to animal modeling, the careful selection of phages for therapeutic applications is especially important. Indeed, it is important to select phages that do not undergo lysogeny and do not carry toxin and antibiotic resistance genes. The selection of phage strains that are resilient to resistance has received little attention, which could be due to the unproven premise that phage cocktails will prevent resistance development [23,87]. Among 12 phage therapy human clinical studies that implemented phage cocktails, 7 cases confirmed the emergence of phage resistance during treatment [74]. For example, *Acinetobacter baumannii* developed resistance to all eight phages used to treat bacteremia after just 1 week of treatment [88]. Another criterion for phage selection that is lacking exploration is phage infection performance under human physiologic hypoxia conditions. Together, the features of CrRp10 suggest that it could be a promising therapeutic agent in mouse models of diarrheal diseases.

## Figures and Tables

**Table 1:** Citrobacter phages and their genome features.

Phage	Host	Source	Phage Family *	Size (kb)	GC%	Accession No.	Ref.
CrRp3	*C. rodentium*	wastewater	A	44.3	45.1	MG775042	This study
CR44b	*C. rodentium*	sewage effluent	A	39.2	50.5	NC_023576	[21]
CR8	*C. rodentium*	sewage effluent	A	39.7	49.7	NC_023548	[21]
CVT22	*Citrobacter* sp.	termite gut	A	47.6	41.6	NC_027988	[51]
phiCFP-1	*C. freundii*	seawater	A	38.6	50.3	NC_028880	N/A
SH1	*C. freundii*	seawater	A	39.4	51	NC_031066	N/A
SH2	*C. freundii*	seawater	A	39.2	50.7	NC_031092	N/A
SH3	*C. freundii*	seawater	A	39.4	50.6	NC_031123	N/A
SH4	*C. freundii*	seawater	A	39.3	52.6	NC_031018	N/A
CrRp10	*C. rodentium*	municipal wastewater	M	171.5	35.5	MG775043	This study
R18C	*C. rodentium*	rabbit feces	M	31.8	51.6	MN016939	[20]
IME-CF2	*C. freundii*	hospital wastewater	M	177.7	43.2	NC_029013	N/A
Margaery	*C. freundii*	wastewater	M	178.2	44.9	NC_028755	N/A
Merlin	*C. freundii*	wastewater	M	172.7	38.8	NC_028857	[52]
Miller	*C. freundii*	wastewater	M	178.2	43.1	NC_025414	[53]
Moon	*C. freundii*	wastewater	M	170.3	38.9	NC_027331	[54]
Michonne	*C. freundii*	wastewater	M	90.0	38.8	NC_028247	[55]
Moogle	*C. freundii*	wastewater	M	88.0	39	NC_027293	[56]
CfP1	*C. freundii*	sewage effluent	M	180.2	43.1	NC_031057	N/A
Stevie	*C. freundii*	soil	D	49.8	42.8	NC_027350	[57]

* *Autographiviridae* (A), *Myoviridae* (M), *Drexlerviridae* (D).

**Table 2:** CrPp3 and CrRp10 replication characteristics on *C. rodentium* strain ICC180.

	CrRp3	CrRp10
Family	*Autographiviridae*	*Myoviridae*
Adsorption constant ^1^ (mL^−1^ min^−1^)	k =3.50 × 10^−10^ (±3.2 × 10^−10^)	k =8.52 × 10^−11^ (±2.8 × 10^−11^)
Latent period ^2^	15 (±2) min	17 (±2) min
Mean burst size	43 (±22) PFU	85 (±16) PFU

^1^ k = 2.3/Bt*log(*P*0 *P*^−1^); t(0) to t(50% bound); ±stdev; ^2^ The latent period represents t(0) to t(cell lysis).

**Table 3:** Phage strain host range.

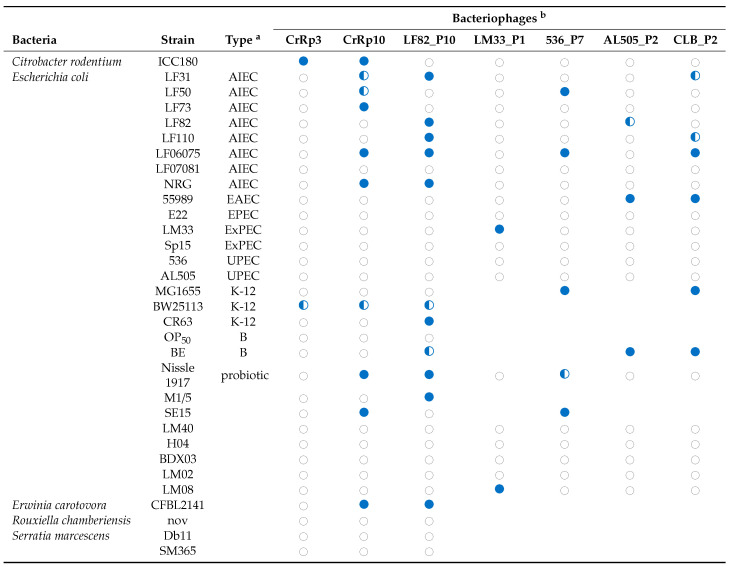									

^a^ AIEC (Adherent Invasive *E. coli*); EAEC (enteroaggregative *E. coli*); EPEC (enteropathogenic *E. coli*); ExPEC (extraintestinal *E. coli*); UPEC (uropathogenic *E. coli*); ^b^ A 4 µL drop of 10^7^ PFU mL^−1^ on a bacterial lawn. Full clearing ●; partial clearing ◐; no clearing ○.

## Supplementary Materials

The following are available online at https://www.mdpi.com/1999-4915/12/7/737/s1, Figure S1: Amino acid sequence alignments to compare the compositions of *Citrobacter* phages (a) podoviruses and (b) myoviruses, Figure S2: One-step growth curves of phage (a) CrRp3 and (b) CrRp10, Table S1: *Citrobacter* phage CrRp3 annotations, Table S2: *Citrobacter* phage CrRp10 annotations, Table S3: Classification of the best hits of phages CrRp3 and CrRp10, Table S4: *E. coli* phage complete genomes used in genome-blast distance phylogeny analyses.
