# Supplementary material for: Isolation and Characterization of Bacteriophages That Infect Citrobacter rodentium, a Model Pathogen for Intestinal Diseases
Source: Viruses. 2020 Jul 8;12(7):737. doi: 10.3390/v12070737 (PMC7412075; doi:10.3390/v12070737)
Supplement: Supplementary file 1 [file viruses-12-00737-s001.zip › Supplementary Fig1.pdf]

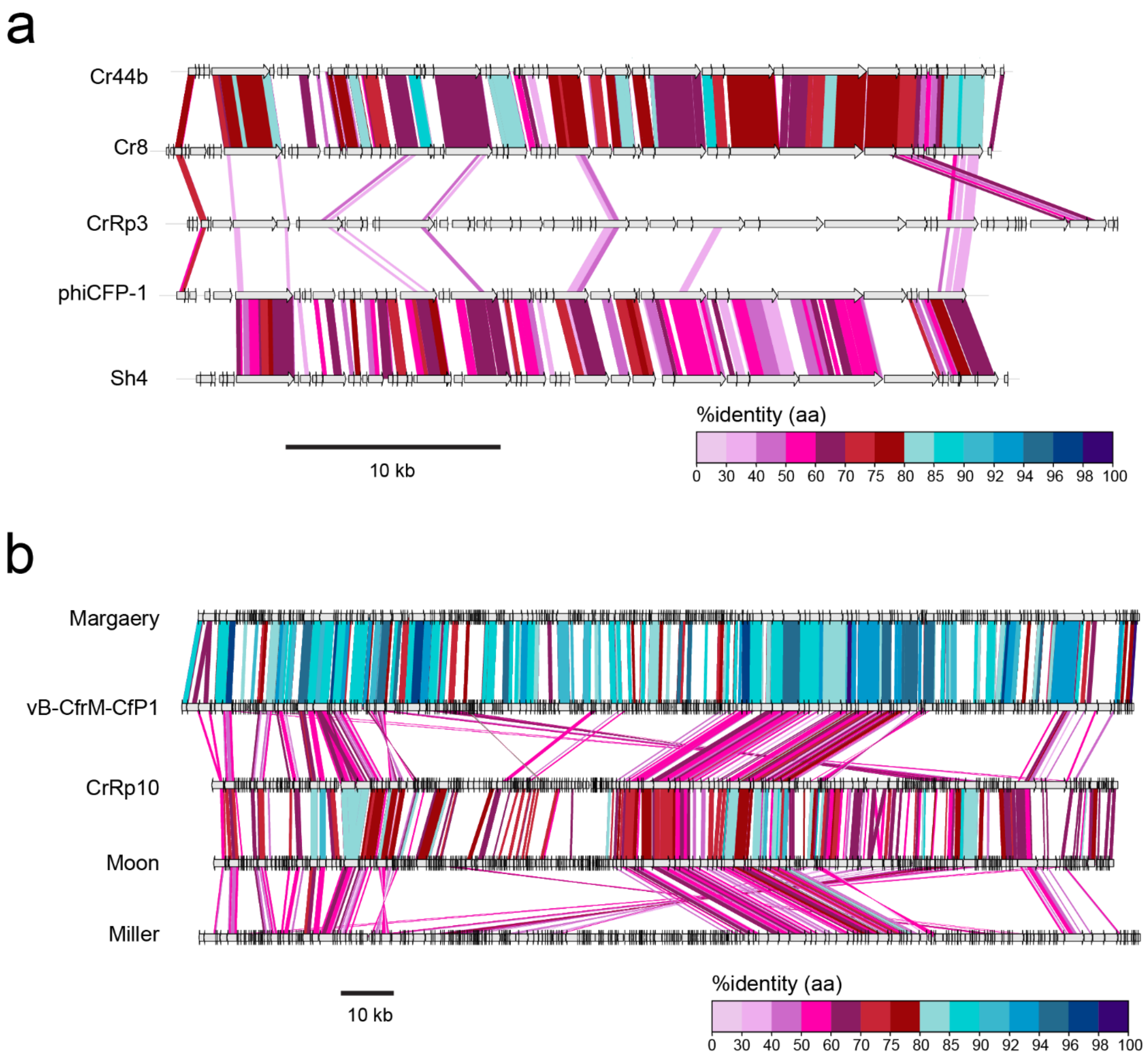

Supplementary Figure 1. Amino acid sequence alignments to compare the compositions of Citrobacter phages (a) podoviruses and (b) myoviruses.
