# Supplementary material for: Isolation and Characterization of Bacteriophages That Infect Citrobacter rodentium, a Model Pathogen for Intestinal Diseases
Source: Viruses. 2020 Jul 8;12(7):737. doi: 10.3390/v12070737 (PMC7412075; doi:10.3390/v12070737)
Supplement: Supplementary file 1 [file viruses-12-00737-s001.zip › Supplementary Fig2.pdf]

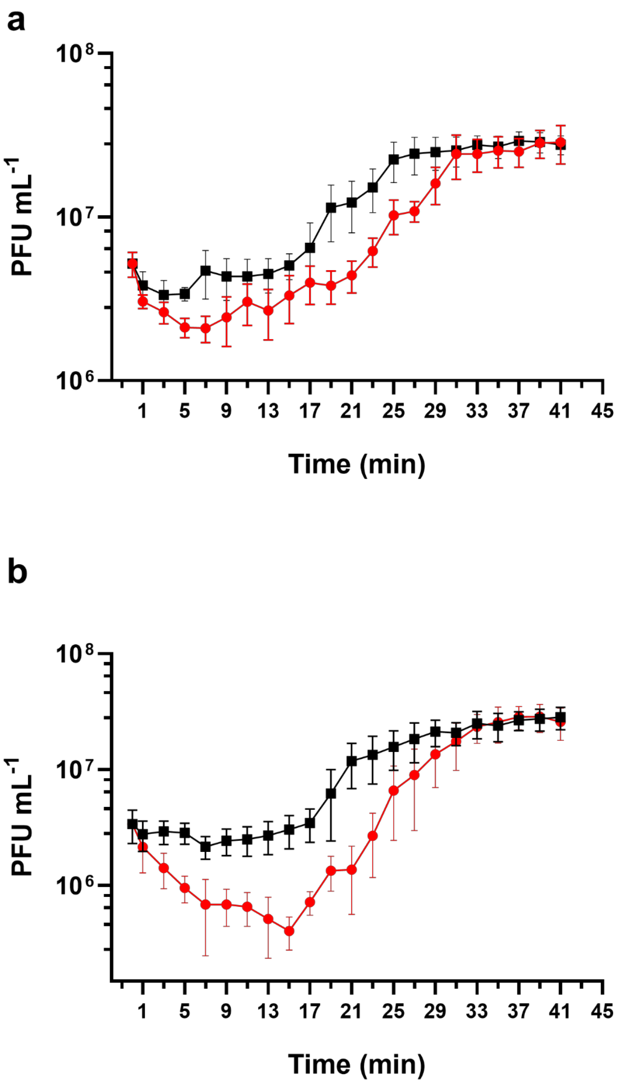

Supplementary Figure 2. One-step growth curves of phage (a) CrRp3 and (b) CrRp10. Phage production post *Citrobacter rodentium* infection at a MOI of 0.01. Titer comparison between chloroform-treated (red) and non-treated (**black**) samples was used to estimate eclipse and latent periods respectively. Although, the eclipse periods could not be estimated, CrRp3 exhibited a latent period of approximately 15 min and **C**rRp10 of 17 min. Error bars represent S.E.M. N=4 per phage.
