# Supplementary figures and images for: Isolation and Characterization of Bacteriophages That Infect Citrobacter rodentium, a Model Pathogen for Intestinal Diseases
Source: Viruses. 2020 Jul 8;12(7):737. doi: 10.3390/v12070737 (PMC7412075; doi:10.3390/v12070737)

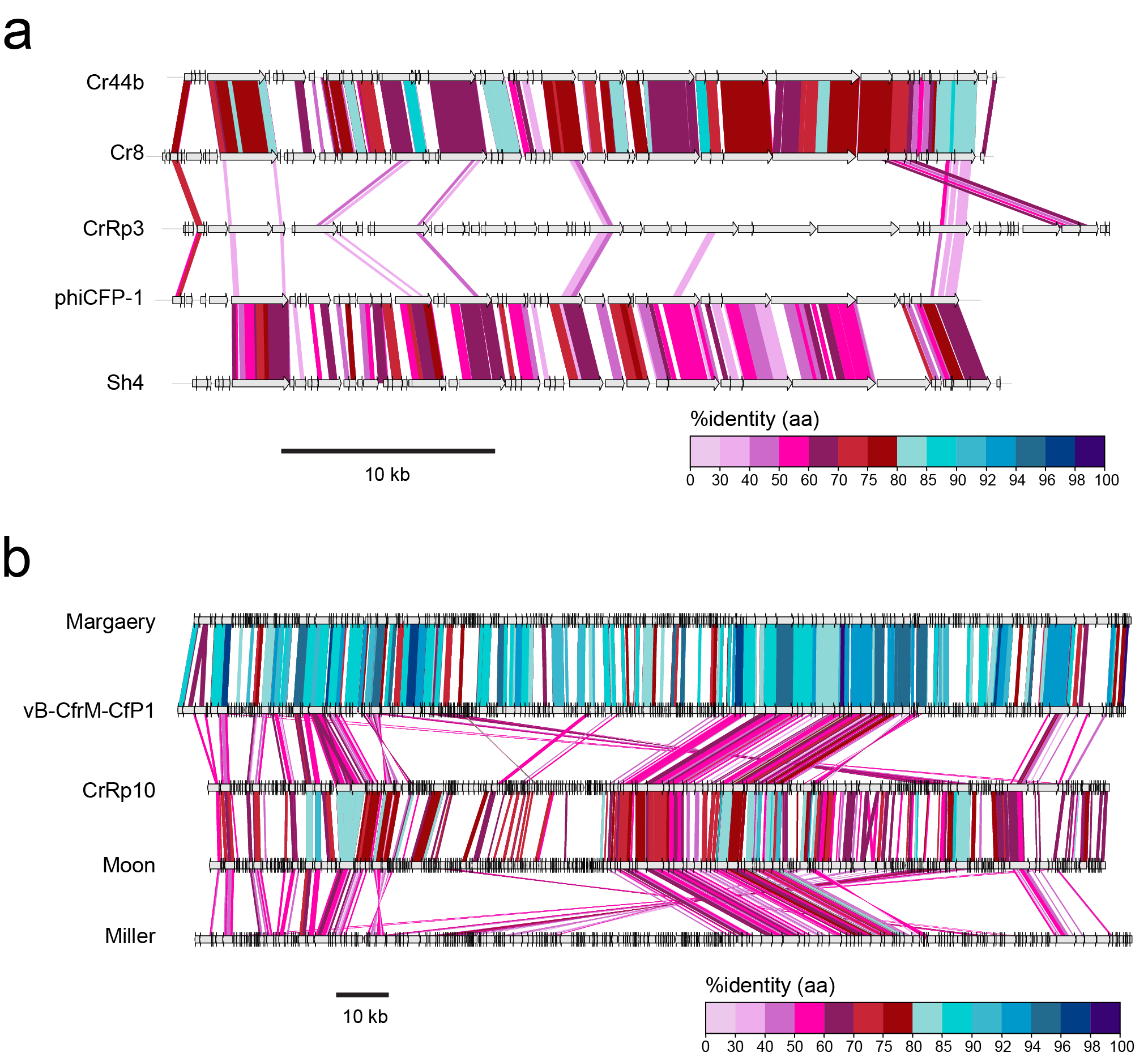

Supplement: Supplementary file 1 [file viruses-12-00737-s001.zip › Supplementary Fig1.tif]

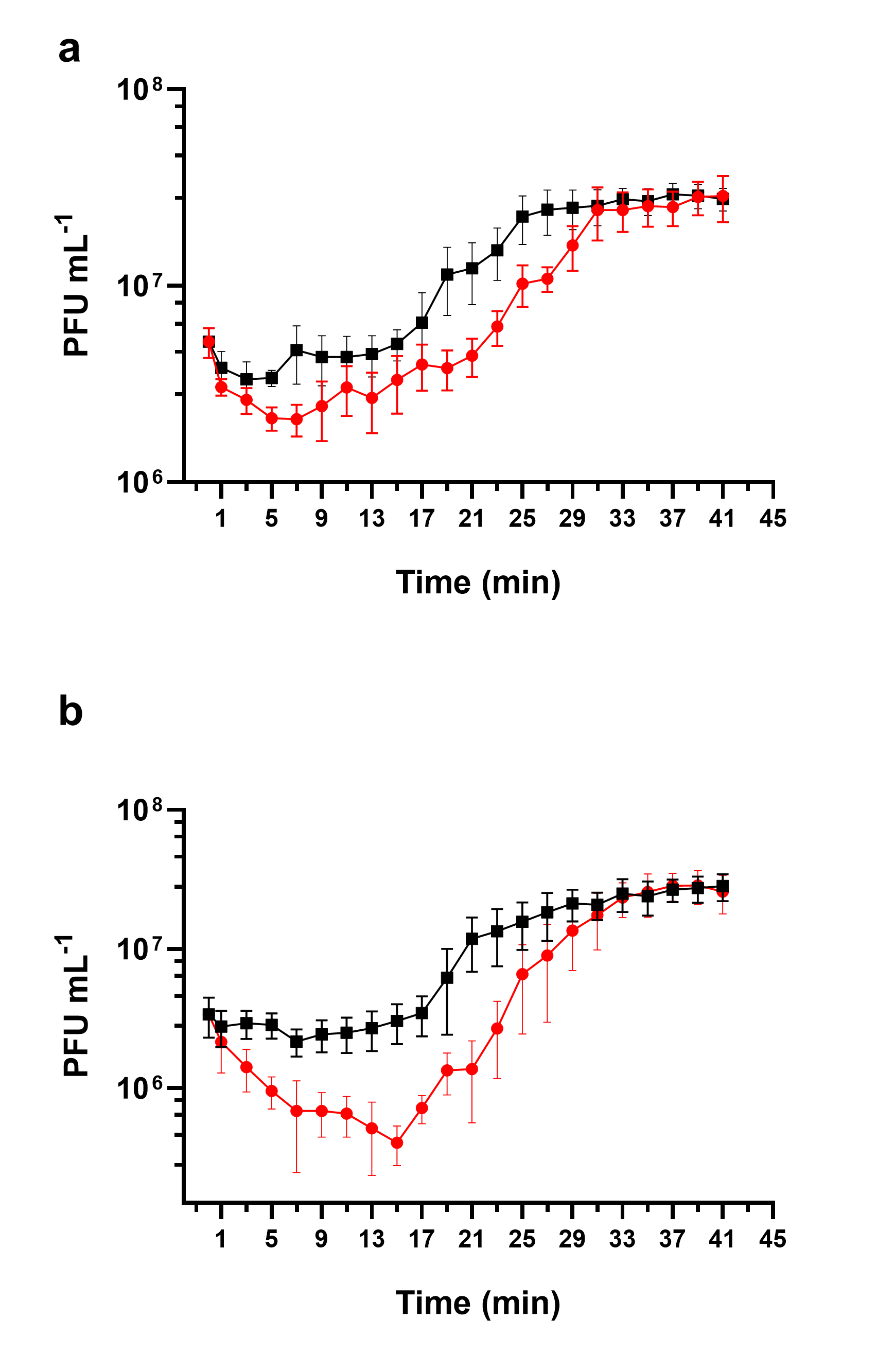

Supplement: Supplementary file 1 [file viruses-12-00737-s001.zip › Supplementary Fig2.tif]
